# Stereotactic body radiotherapy for single and multiple early-stage non-small cell lung cancer in patients aged ≥ 80 years

**DOI:** 10.1186/s13014-025-02693-w

**Published:** 2025-07-17

**Authors:** Samuel M. Vorbach, Thomas Seppi, Jan C. Peeken, Manuel Sarcletti, Martin Pointner, Katharina Hörmandinger, Julian Mangesius, Meinhard Nevinny-Stickel, Ute Ganswindt

**Affiliations:** 1 Department of Radiation Oncology, Medical University of Innsbruck, Innsbruck, Austria; 2 Department of Radiation Oncology, Klinikum rechts der Isar, Technical University of Munich (TUM), Munich, Germany; 3 German Consortium for Translational Cancer Research (DKTK), Partner Site Munich, Munich, Germany; 4 Institute of Radiation Medicine (IRM), Helmholtz Zentrum München (HMGU), Neuherberg, Germany

**Keywords:** Stereotactic body radiotherapy, Lung cancer, Elderly, Multiple primary lung cancer

## Abstract

**Background:**

Lung cancer primarily affects elderly individuals and is the leading cause of cancer-related death in people aged 80 years and older. In addition, the incidence of multiple primary lung cancer (MPLC) is increasing worldwide. Although surgery is recommended as the standard of care, many elderly patients are considered medically unsuitable, or they refuse surgery. The role of stereotactic body radiotherapy (SBRT) as an alternative treatment option for these elderly patients, particularly those with multiple primary lung cancer, has not been fully elucidated. Therefore, the aim of this study was to report the outcome and toxicities associated with SBRT for histologically confirmed early-stage non-small cell lung cancer (NSCLC) and synchronous and metachronous multiple primary lung cancer in patients aged ≥ 80 years.

**Methods:**

This retrospective study included 118 patients aged ≥ 80 years with a total of 141 SBRT-treated primary lung cancers (19 patients with MPLC). We assessed local control (LC), progression-free survival (PFS), overall survival (OS) and cancer-specific survival (CSS). We further evaluated toxicities and factors impacting therapeutic efficacy.

**Results:**

The median follow-up after SBRT was 47 months (range 3–169 months). The LC rate was 96.2% (95% CI: 90.1 to 98.6%) two years and 86.4% (71.8 to 93.8%) five years after SBRT for NSCLC/MPLC. The PFS and OS rates were 67.0% (57.4 to − 74.9%) and 74.7% (65.4 to − 81.1%), respectively, after two years and 24.7% (14.5 to 35.6%) and 30.2% (19.4 to 41.7%), respectively, after five years. The CSS rate was 88.6% (80.3–93.6%) at two years and 76.6% (61.4–86.4%) at 5 years after SBRT. Age and the Charlson Comorbidity Index score were found to be independent predictors of OS and PFS. Predictors other than these patient-related factors could not be identified. Toxicities higher than Grade 2 after SBRT of NSCLC and MPLC were not observed.

**Conclusion:**

This study emphasises the efficacy and safety of SBRT in the treatment of early-stage NSCLC in patients aged ≥ 80 years, including those with MPLC. SBRT proves to be an appropriate treatment modality for this frail patient group, as it provides favourable LC and CSS rates with low toxicity.

## Background

Worldwide, almost one in five (18.4%) cancer deaths are caused by lung cancer, which is still the most common cause of cancer death worldwide. The number of cases continues to increase, particularly in Asia, which is home to almost 60% of the world’s population [1]. This trend is expected to continue in the future, as the ageing population in many populous nations is constantly increasing. As a consequence, an increasing number of people have surpassed the median age of diagnosis of lung cancer (71 years). More than one-third of patients diagnosed with non-small cell lung cancer (NSCLC) are older than 75 years of age [2]. Surgery is recommended as the standard treatment for early-stage NSCLC, even for elderly patients, yielding overall cancer-related survival rates comparable to those reported in patients younger than aged 70 years [3, 4]. Nevertheless, elderly individuals are more likely to refuse surgery [5] or be deemed medically unsuitable due to predominant comorbidities or impaired physical status. In addition, surgery has been associated with high morbidity and mortality in elderly patients aged ≥ 80 years [6]. With a local control (LC) rate of approximately 90%, stereotactic body radiotherapy (SBRT) is the standard of care for medically inoperable early-stage NSCLC and is associated with low toxicity [7–9]. Furthermore, survival rates after SBRT are generally considered comparable to those for surgery [9–11]. Moreover, SBRT is becoming increasingly important as an alternative primary treatment strategy for elderly individuals.

In addition, the incidence of multiple primary lung cancer (MPLC) is increasing worldwide [12]. MPLC can occur synchronously (sMPLC) or metachronously (mMPLC) depending on the time of diagnosis. Despite a longer history of establishing MPLC diagnoses over the last five decades [13, 14], the definition of diagnostic criteria for sMPLC and mMPLC is still not universally standardized due to the lack of authoritative guidelines. In particular, the distinction between MPLC and intrapulmonary metastases (IPM) of lung cancer is crucial for reliable prognosis and appropriate treatment selection [15], but it is also subject to considerable uncertainty, mainly due to similar histologies, which have been reported to be present in more than half of the diagnosed MPLCs [16, 17]. Potential genomic indicators and biomarkers are currently being investigated to ensure that MPLC and IPM can be differentiated in the future with a lower rate of misdiagnosis [14]. Notwithstanding the persisting diagnostic uncertainties, surgery remains the preferred treatment approach for MPLC, with SBRT being a viable alternative [14].

This retrospective, single-centre study aims to evaluate the long-term clinical outcomes, prognostic factors, and toxicities of SBRT in patients aged ≥ 80 years with histologically confirmed early-stage NSCLC, including those with synchronous and metachronous MPLC. To our knowledge, this is the first study to systematically report SBRT outcomes in a specific high-risk subpopulation with multiple primaries based on a homogeneous cohort with pathological confirmation and long follow-up. By investigating whether excellent local control and cancer-specific survival can be achieved even for patients with MPLC, this study also provides novel evidence for the safe and effective application of SBRT in this frail patient cohort, thereby expanding the evidence base for personalised therapy in this growing demographic population.

## Methods

### Study population

Patients aged ≥ 80 years at the start of SBRT for histologically confirmed NSCLC (T1-T2N0M0), who were treated at the Department of Radiation Oncology, Medical University of Innsbruck in Austria between December 2007 and December 2023 were retrospectively identified. All patients underwent imaging-based staging. In 96% of the patients, positron emission tomography/computed tomography (PET/CT) was used, while the remaining patients received contrast-enhanced computed tomography (CT) scans alternatively. Tumours were pathologically confirmed by CT-guided or bronchoscopic biopsy. The distinction between IPM and multiple primary lung cancers was made by the institutional multidisciplinary tumour board. A lung nodule diagnosed in addition to the primary tumour was classified as a separate primary if it exhibited a different histologic subtype or if it exhibited the same histology but was located in a different lobe with no evidence of metastatic spread to the lymph nodes or elsewhere. All patient cases were discussed with the institutional multidisciplinary tumour board. Patients included in this study were either defined as medically unsuitable for surgery based on pre-treatment pulmonary function tests, comorbidities and general condition, or they declined surgery.

Demographic factors, including age in years, sex and smoking history, were recorded at the time of SBRT. The Eastern Cooperative Oncology Group (ECOG) performance score was used to assess patients’ functional status and ability to provide self-care [18]. The age-adjusted Charlson Comorbidity Index [19] was calculated to more precisely assess the burden of comorbidities. All patients met the following criteria: (I) aged ≥ 80 years on the first day of SBRT; (II) no history of radiation therapy or surgery involving the lung; and (III) no other active cancers.

### Techniques of radiotherapy

Elekta BodyFIX was used to immobilise patients in the supine position. Starting in 2012, free-breathing four-dimensional CT scans were acquired to capture the location and movement of the tumour over time. Image fusion with PET/CT scans was performed to assist in the delineation of target volumes. The gross tumour volume (GTV) was contoured in the lung window range. GTV with consideration of tumour motion was used to create an internal target volume (ITV). The creation of a planning target volume (PTV) involved expanding the ITV by 4–8 mm.

Tumours were classified as central (within 2 cm of any critical mediastinal structure in all directions, including the bronchial tree, oesophagus, heart, brachial plexus, major vessels, spinal cord, phrenic nerve, and recurrent laryngeal nerve) [20] or ultracentral (defined in the SUNSET trial as a tumour in which the PTV touches or overlaps the central bronchial tree, oesophagus, pulmonary vein, or pulmonary artery) [21]. Any tumours that did not meet the criteria for the central or ultracentral definitions were classified as peripheral. Different dose concepts were applied depending on the tumour location, PTV size and discretion of the treating radiation oncologist: 60 Gy in 10 fractions for central and ultracentral tumours (prescribed to the 100% isodose), 48 Gy in 6 fractions for central tumours, and 48 Gy in 4 fractions or 45 Gy in 3 fractions for peripheral tumours (all prescribed to the 65% isodose). SBRT was delivered using the free-breathing technique.

Treatment planning was performed using precisePLAN (Elekta AB, Stockholm Sweden) until 2013 and Pinnacle Software (most recent version V14; Philips Medical, Fitchburg, USA) until the end of the study. Patients were treated using three-dimensional conformal radiation therapy or volumetric modulated arc therapy using an Elekta Synergy linear accelerator until 2013 and then with a Versa HD linear accelerator (both from Elekta AB, Stockholm, Sweden). Image guidance was performed with daily cone beam CT scans.

### Follow-up

After the completion of SBRT, follow-up radiological imaging (usually CT scan, if necessary PET/CT) was performed every 3 months for 1.5 years and then every 6 months thereafter. All cases in which recurrence was suspected were evaluated by the interdisciplinary tumour board. Tumour response was classified by two independent radiologists with more than 15 years of experience, according to RECIST (Response Evaluation Criteria in Solid Tumours) [22]. The follow-up time was defined as the time between the end of SBRT and the last follow-up date. LC was defined as progression of the treated lesion and was measured as the time from the end of SBRT to progression or to the last follow-up. Progression-free survival (PFS) was defined as the time from the end of SBRT to the first occurrence of either disease progression (in the treatment field or outside) or death from any cause. Patients without progression or death were censored at the date of the last follow-up. Overall survival (OS) was defined as the time from the end of SBRT to either death from any cause or to the date of the last follow-up. Cancer-specific survival (CSS) was defined as the time from the end of SBRT to death from lung cancer. Toxicity was monitored by clinical follow-up, laboratory testing and medical imaging and classified according to the Common Terminology Criteria for Adverse Events (CTCAE version 3.0–5.0 [23]).

### Statistical analysis

The endpoints of this study were LC rate, PFS, OS and CSS. Statistical analysis was conducted using SPSS Statistics (V26, IBM Cooperation, Armonk, NY, USA) and GraphPad Prism (V10, GraphPad Software Inc., San Diego, CA, USA). Descriptive analysis was used to summarise the relevant patient and treatment characteristics. LC, PFS, OS and CSS were calculated using the Kaplan-Meier method. The median follow-up was calculated using the reverse Kaplan method. The Cox proportional hazards model was used for univariate and multivariate analyses of the factors and their hazard ratios with 95% confidence intervals associated with LC, PFS, OS and CSS. Multivariate analysis was performed by applying the rule of stepwise backwards elimination of nonsignificant factors. Differences in the frequency of toxicities were assessed using Fisher’s exact test. P values < 0.05 were considered to indicate statistical significance. The linear quadratic model was used to calculate biologically effective doses (BEDs) for all radiotherapy prescription doses with an assumed alpha/beta ratio of 10 (BED10).

## Results

### Patient population

Patient data, lung cancer specifications and treatment characteristics are summarized in Table 1. This study included 118 patients, all aged ≥ 80 years (median age 82 years, range 80–91 years), with pathologically proven NSCLC at the time of SBRT. Among these, 73 (61.9%) patients were males, and the remaining 45 were females. The median ECOG performance status of all patients was 1 (range 0−2). The age-adjusted Charlson Comorbidity Index (4 index points added for an age ≥ 80 years) ranged from 4−12, with the majority of patients (79%) exhibiting a lower comorbidity score between 4 and 6. Our cohort consisted of 44.9% of patients without any smoking history. Smokers and former smokers had a median of 22 pack years (range 3–65). The vast majority (86.4%) of patients were classified as unsuitable for surgery by the interdisciplinary tumour board. The remaining 16 patients (13.6%) rejected the recommended surgery and subsequently presented for SBRT. Among all patients, 83.9% were treated upon detection of one early-stage primary tumour (*n* = 99).

A total of 15 patients (12.7%) were treated for two primary tumours (10 synchronous vs. 5 metachronous), and 4 patients (2.4%) received SBRT for three primary lung cancers (2 synchronous vs. 2 synchronous as well as metachronous). Metachronously diagnosed MPLC patients were treated at a mean interval of 24.0 months (SD 17.4 months).

To summarise, a total of 118 patients with 141 lung primaries were treated with SBRT. Pathology revealed 70.9% adenocarcinoma (*n* = 100) and 29.1% squamous cell carcinoma (*n* = 41)..

**Table 1:** Patient characteristics, lesions and treatment

Characteristic		Value or no. (%)
Total no. of patients		118
Total no. of primary lung cancers		141
Age at start or SBRT	Median	82
	Range	80-91
Sex	Male	73 (61.9%)
	Female	45 (38.1%)
ECOG Performance Status	0	49 (41.5%)
	1	60 (50.8%)
	2	9 (7.6%)
Charlson Comorbidity Index	4	30 (25.4%)
	5	35 (29.7%)
	6	27 (22.9%)
	7	10 (8.5%)
	8	5 (4.2%)
	9	7 (5.9%)
	10	3 (2.5%)
	12	1 (0.8%)
Smoking history	Yes	65 (55.1%)
	No	53 (44.9%)
Pack years of smokers	Median	22
	Range	3-65
Operability	Yes (refused)	16 (13.6%)
	No	102 (86.4%)
Number of primary lung cancers treated with SBRT per patient (metachronous and synchronous)	1	99 (83.9%)
	2	15 (12.7%)
	3	4 (2.4%)
Histology of lung cancers	Adenocarcinoma	100 (70.9%)
	Squamous cell carcinoma	41 (29.1%)
AJCC tumour classification (valid at the time of diagnosis)	T1	102 (72.3%)
	T2	39 (27.7%)
Location	Upper lobe	85 (60.3%)
	Middle lobe	4 (2.8%)
	Lower lobe	52 (36.9%)
Location	Peripheral	101 (71.6%)
	Central	35 (24.8%)
	Ultracentral	5 (3.5%)
PTV (cm³)	Median	37.40
	Range	5.4–186.0
Single dose prescribed (PTV encompassing, Gy)	Median	15
	Range	6-15
Total dose prescribed (PTV encompassing, Gy)	Median	45
	Range	45-60
BED10 at PTV periphery (Gy)	Median	112.5
	Range	86.4–112.5

AJCC: American Joint Committee on Cancer

### Treatment and outcomes

The median PTV was 37.4 (range 5.4−186.0) cm³. Dose prescriptions included 3 × 15 Gy (*n* = 72; 51.1%), 4 × 12 Gy (*n* = 7; 5.0%), 10 × 6 Gy (*n* = 25; 17.7%), and 6 × 8 Gy (*n* = 37; 26.2%). The median BED10 at the PTV periphery was 112.5 Gy (range 86.4–112.5 Gy).

**Fig. 1:**
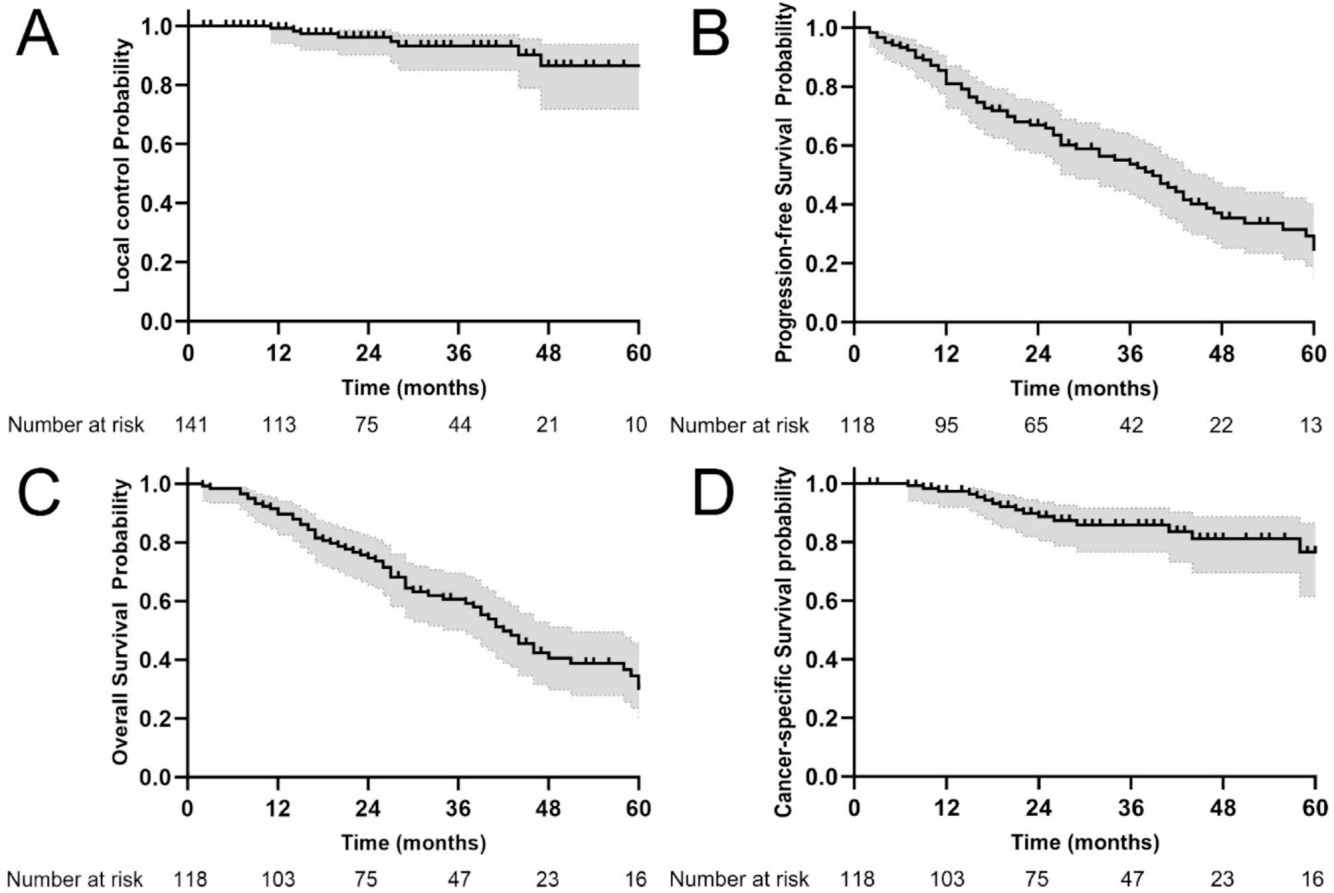
Kaplan-Meier curves showing **(a)** local control (141 SBRT-treated NSCLC) **(b)** progression-free survival, **(c)** overall survival, and **(d)** cancer-specific survival for 118 patients aged ≥ 80 years (grey shaded area: 95% confidence intervals)

The median follow-up after SBRT was 47 months (range 3–169 months). The median OS was 42 months (95% CI: 36.2 to 47.8 months). Among all the patients who underwent SBRT, the one-year LC rate, PFS and OS were 99.1% (95% CI: 94.0 to 100.0%), 80.9% (72.5 to 87.0%) and 89.7% (82.5 to 94.0%), respectively, and the two-year LC rate, PFS and OS were 96.2% (90.1 to 98.6%), 67.0% (57.4 to 74.9%) and 74.7% (65.4 to 81.1%), respectively. Five years after SBRT, the five-year PFS and OS rates were 86.4% (71.8 to 93.8%), 24.7% (14.5 to 35.6%) and 30.2% (19.4 to 41.7%), respectively. The CSS rate was 97.3% (91.9 to 99.1%) at one year, 88.6% (80.3 to 93.6%) at two years and 76.6% (61.4 to 86.4%) at 5 years. Kaplan-Meier plots for LC, PFS, OS and CSS are provided in Fig. 1. The clinical outcomes of the MPLC subgroup are summarised in Table 2. In addition, a cancer-specific survival rates reported in international studies are summarised and compared to our data in Table 3.

**Table 2:** Outcomes of patients treated for multiple primary lung cancer

	At one year (95% confidence interval)	At two years (95% confidence interval)	At five years (95% confidence interval)
Local control rate (%)	100.0 (100.0-100.0)	94.2 (78.7–98.5)	69.6 (38.6–88.1)
Progression-free survival rate (%)	78.9 (53.2–91.5)	66.8 (40.3–83.6)	34.3 (10.9–58.0)
Overall survival rate (%)	89.5 (64.1–97.3)	77.1 (49.6–90.8)	38.5 (7.9–69.8)
Cancer-specific survival rate (%)	94.7 (68.1–99.2)	94.7 (68.1–99.2)	55.3 (8.4–86.9)

**Table 3:** Summary of cancer-specific survival rates in patients with early-stage NSCLC treated with SBRT

		Cancer-specific survival rate (%) at		
Study	**One year**	**Two years**	**Three years**	**Five years**
Cassidy et al. [24]	96*	81.6	72.6	72*
Takeda et al. [25]	99.1	86.2	70.8	95
Bei et al. [26]	98*	93*	75.7	62*
Watanabe et al. [6]	98.4	98.4	93.7	83.5
Aoki et al. [5]	97*	93.1	87.5	70*
Present study	97.3	88.6	85.8	76.6

* estimated data derived from interpolation of published Kaplan-Meier curves

In total, seven patients experienced tumour recurrence (out of 141) at the SBRT-treated site, with a median time to recurrence of 26 months (range 14–47 months). Among these seven patients, three died with local and distant tumour progression, one patient experienced local progression but died because of fulminant SCLC, one patient was lost to follow-up one year after local recurrence, and two patients experienced local progression without distant metastases and died due to cerebral haemorrhage.

A total of 66 patients died, but only 17 of whom had progressive tumour disease. Death of the remaining 49 patients were due to the following: other malignancies (4), heart failure (1), pulmonary embolism (1), acute myocardial infarction (3), gastrointestinal bleeding (3), cerebral haemorrhage (2), urosepsis (3), severe pneumonia (10), and unknown causes but no history of tumour progression (22).

In the univariate analysis, the prognostic factors significantly associated with reduced PFS were age (HR = 1.12, 95% CI: 1.02–1.22, *p* = 0.012; see Table 4) and a higher Charlson comorbidity index (≥ 7 vs. 4–6: HR = 2.23, 95% CI: 1.29–3.85, *p* = 0.004). The multivariate analysis confirmed that both factors were significantly associated with reduced PFS: age (HR = 1.13, 95% CI: 1.03–1.23, *p* = 0.008) and a higher Charlson Comorbidity Index (≥ 7 vs. 4–6, HR = 2.33, 95% CI: 1.34–4.03, *p* = 0.003).

**Table 4:** Univariate and multivariate analyses of risk factors related to PFS

Univariate Hazard Ratio for PFS			Multivariate Hazard Ratio for PFS	
Factor	**HR (CI 95%)**	*p Value*	**HR (CI 95%)**	*p Value*
Sex				
Male	1 (reference)			
Female	0.81 (0.50–1.32)	0.403		
Age	1.12 (1.02-1.22)	**0.012**	1.13 (1.03-1.23)	**0.008**
Charlson Comorbidity Index				
4-6	1 (reference)		1 (reference)	
≥7	2.23 (1.29–3.85)	**0.004**	2.33 (1.34-4.03)	**0.003**
Number of primary lung cancer				
1	1 (reference)			
≥2	1.03 (0.54–1.98)	0.922		

In the univariate analysis, both age (HR = 1.16, 95% CI: 1.06–1.27, *p* = 0.010) and a higher Charlson Comorbidity Index (≥ 7 vs. 4–6, HR = 2.34, 95% CI: 1.33–4.11, *p* = 0.003; see Table 5) were associated with shorter OS. In the multivariate analysis, both age (HR = 1.17, 95% CI: 1.07–1.28, *p* < 0.001) and a higher Charlson Comorbidity Index (≥ 7 vs. 4–6, HR = 2.44, 95% CI: 1.38–4.32, *p* = 0.003) were confirmed as prognostic factors significantly associated with impaired OS.

The Cox proportional hazards model for CSS did not identify any significant predictors (see Table 6). No significant association with local control was observed in the univariate analysis for histology (adenocarcinoma vs. squamous cell carcinoma, *p* = 0.272), PTV (*p* = 0.395) or BED₁₀ (> 100 Gy vs. <100 Gy, *p* = 0.846).

**Table 5:** Univariate and multivariate analyses of risk factors related to OS

Univariate Hazard Ratio for OS			Multivariate Hazard Ratio for OS		
Factor	**HR (CI 95%)**	*p Value*		**HR (CI 95%)**	*p Value*
Sex					
Male	1 (reference)				
Female	0.96 (0.58-1.58)	0.869			
Age	1.16 (1.06-1.27)	**0.010**		1.17 (1.07-1.28)	**<0.001**
Charlson Comorbidity Index					
4-6	1 (reference)			1 (reference)	
≥7	2.34 (1.33-4.11)	**0.003**		2.44 (1.38-4.32)	**0.003**
Number of primary lung cancer					
1	1 (reference)				
≥2	0.73 (0.35-1.53)	0.400			

**Table 6:** Univariate analysis of risk factors related to CSS

Univariate Hazard Ratio for CSS		
Factor	**HR (CI 95%)**	*p Value*
Sex		
Male	1 (reference)	
Female	1.36 (0.52-3.55)	0.526
Age	1.15 (0.97-1.36)	0.119
Charlson Comorbidity Index		
4-6	1 (reference)	
≥7	1.57 (0.50-4.94)	0.438
Number of primary lung cancer		
1	1 (reference)	
≥2	1.56 (0.51-4.79)	0.439

### Treatment-related toxicities

SBRT was well tolerated, despite the advanced age of the patients and the high rate of comorbidities, and all patients completed their SBRT courses as planned. None of the patients experienced SBRT-induced lung toxicity ≥ grade 3 until the last follow-up visit. In total, 19 patients (16.1%) developed grade 2 pneumonitis requiring steroids. The median time to onset of pneumonitis was 4.4 months (range 1.0–7.1 months) after the completion of SBRT. Fisher’s exact test revealed that the frequency of pneumonitis did not differ significantly between patients treated for one NSCLC and patients treated for MPLC (*p* = 0.162, one-tailed). In 12 patients (10.2%), rib fractures were detected by CT imaging during oncological follow-up. These patients did not report any symptoms caused by their rib fractures, and they did not present to clinics with pain. The fractures were already consolidated in all patients at the time of diagnosis. None of these patients had previously been treated for MPLC. No other SBRT-associated toxicities ≥ grade 3 (e.g., oesophagitis, fatigue, stenosis or haemorrhage) were detected.

## Discussion

Our findings support SBRT as a compelling alternative to surgical resection for early-stage NSCLC in elderly patients aged ≥ 80 years, offering excellent local control, robust CSS, and a very favourable toxicity profile. Notably, this is the first study to report outcome data on a larger subcohort of octogenarians (*N* = 19) treated with SBRT for both synchronous and metachronous MPLC.

Remarkable local control rates were achieved, reaching 99.1% after one year, 96.2% after two years, and 86.4% after five years. These results are consistent with those of prior studies of SBRT-treated NSCLC in octogenarians, such as those by Watanabe et al. [6] and Kreinbrink et al. [24], both of which reported excellent LC outcomes. Our analysis did not identify histology, PTV or BED₁₀ as predictors of LC, which echoes the findings of Watanabe et al. [6] but diverges from those of Cassidy et al. [25], who reported an influence of performance status, tumour size, and squamous histology. Importantly, Cassidy’s cohort included patients without histological confirmation and tumours staged up to T3, which complicates direct comparisons.

PFS at one and two years was 80.9% and 67.0%, respectively. Our cohort data aligns closely with data from Takeda et al. [26] and exceeds that of Kreinbrink et al. [24]. By five years, the PFS had decreased to 24.7%, reflecting the expected impact of age-related noncancer mortality: of the 66 total deaths, only 17 were cancer related. The influence of age and the Charlson Comorbidity Index, both patient-related factors, as significant predictors of PFS underscores the predominance of baseline frailty over tumour biology, as indicated by Cassidy et al. [25] and Watanabe et al. [6].

The 1-, 2- and 5-year OS rates for our cohort (89.7%, 74.7%, and 30.2%, respectively) are comparable with those from two American studies [24, 25] but lower than those from Japan [5, 6, 26, 27], where the longer life expectancy may explain the improved long-term OS. Indeed, 41.5% of our patients died from noncancer causes, compared with only 8.4% in the Japanese study by Bei et al. [27]. To eliminate the potential bias of longer life expectancy when comparing the outcomes of patient cohorts aged ≥ 80 years treated in European and North American centres, examining CSS may be appropriate. CSS, indeed, was remarkably consistent across all the studies and countries reviewed (see Table 3 for data summaries), indicating robust tumour control regardless of population differences in longevity.

These findings are further supported by a large, real-world population-based study by van Rossum et al. [28], which evaluated over 7,000 patients with stage I NSCLC treated with SBRT across the Netherlands. Despite the mean patient age of 72.5 years, with over one-fifth of patients aged 80 years or over, the study reported low rates of acute toxicity (3.8%) and 90-day mortality (1.7%). Age was not found to be an independent predictor of either outcome. Instead, performance status and pulmonary function were the strongest determinants of toxicity and short-term mortality risk. This is in line with our own findings that age alone should not be considered a limiting factor when offering SBRT to elderly patients. The authors developed and internally validated clinical prediction models to aid estimation of individual risk, further emphasising the safety and appropriateness of SBRT, even for frail or patients significantly advanced in age, providing that treatment decisions are guided by functional status rather than chronological age.

The treatment tolerance was excellent. No grade ≥ 3 toxicities occurred, and grade 2 pneumonitis was noted in only 16.1% of the patients, with no significant difference between single and multiple SBRT treatments. Rib fractures were asymptomatic and were discovered incidentally. These outcomes reaffirm the safety of SBRT in patients significantly advanced in age, even when used repeatedly for MPLC, as suggested by Griffioen et al. [15].

Several limitations of our study must be acknowledged. Its retrospective design and single-institution scope introduce the potential for selection bias and limit generalisability. In addition, the number of available patients aged ≥ 80 years and treated for MPLCs is by nature restricted, thereby potentially limiting the statistical power to detect significant associations. Nonetheless, this study adds important evidence supporting SBRT in a vulnerable yet growing patient population.

## Conclusions

Our findings reaffirm SBRT as an effective and well-tolerated treatment option for patients aged ≥ 80 years with early-stage NSCLC, including those with multiple synchronous or metachronous primary tumours. The treatment consistently yields high rates of LC and CSS, even in patients requiring repeated SBRT. Given the difficulty in determining the optimal therapeutic approach for the very elderly, our results further support SBRT as a viable and appropriate modality — offering excellent local control rates and a favourable toxicity profile for this challenging cohort.

## Data Availability

The datasets generated and/or analyzed in the current study are not publicly available due to Institutional Review Board requirements, but are available from the corresponding author upon reasonable request.

## Abbreviations

SBRT: Stereotactic body radiotherapy
MPLC: Multiple primary lung cancer
NSCLC: Non-small cell lung cancer
LC: Local control
PFS: Progression-free survival
OS: Overall survival
CSS: Cancer-specific survival
sMPLC: synchronous multiple primary lung cancer
mMPLC: metachronous multiple primary lung cancer
IPM: Intrapulmonary metastases
ECOG: Eastern Cooperative Oncology Group
CT: Computed tomography
PET/CT: Positron emission tomography/computed tomography
GTV: Gross tumour volume
ITV: Internal target volume
PTV: Planning target volume
3D-CRT: Three-dimensional conformal radiation therapy
VMAT: Volumetric modulated arc therapy
RECIST: Response evaluation criteria in solid tumours
CTCAE: Common terminology for adverse events
BED: Biologically effective dose
AJCC: American Joint Committee on Cancer
HR: Hazard ratio
CI: Confidence interval
